# Positions and covering: A two-stage methodology to obtain optimal solutions for the 2d-bin packing problem

**DOI:** 10.1371/journal.pone.0229358

**Published:** 2020-04-06

**Authors:** Nestor M. Cid-Garcia, Yasmin A. Rios-Solis

**Affiliations:** 1 Laboratorio Nacional de Geointeligencia, CONACYT-Centro de Investigación en Ciencias de Información Geoespacial, Aguascalientes, Aguascalientes, Mexico; 2 Escuela de Ingeniería y Ciencias, Tecnológico de Monterrey, Monterrey, Nuevo León, Mexico; 3 Posgrado en Ingeniería de Sistemas, Universidad Autónoma de Nuevo León, Monterrey, Nuevo León, Mexico; University of Hong Kong, HONG KONG

## Abstract

We present a two-stage methodology called *Positions and Covering* (P&C) to solve the two-dimensional bin packing problem (2D-BPP). The objective of this classical combinatorial NP-hard problem is to pack a set of items (small rectangles) in the minimum number of bins (larger rectangles). The first stage is the key-point of the *Positions and Covering*, where for each item, it is generated in a pseudo-polynomial way a set of valid positions that indicate the possible ways of packing the item into the bin. In the second stage, a new set-covering formulation, strengthen with three sets of valid inequalities, is used to select the optimal non-overlapping configuration of items for each bin. Experimental results for the P&C method are presented and compared with some of the best algorithms in the literature for small and medium size instances. Furthermore, we are considering both cases of the 2D-BPP, with and without rotations of the items by 90°. To the best of our knowledge, this is one of the first exact approaches to obtain optimal solutions for the rotation case.

## Introduction

**Fig 1:**
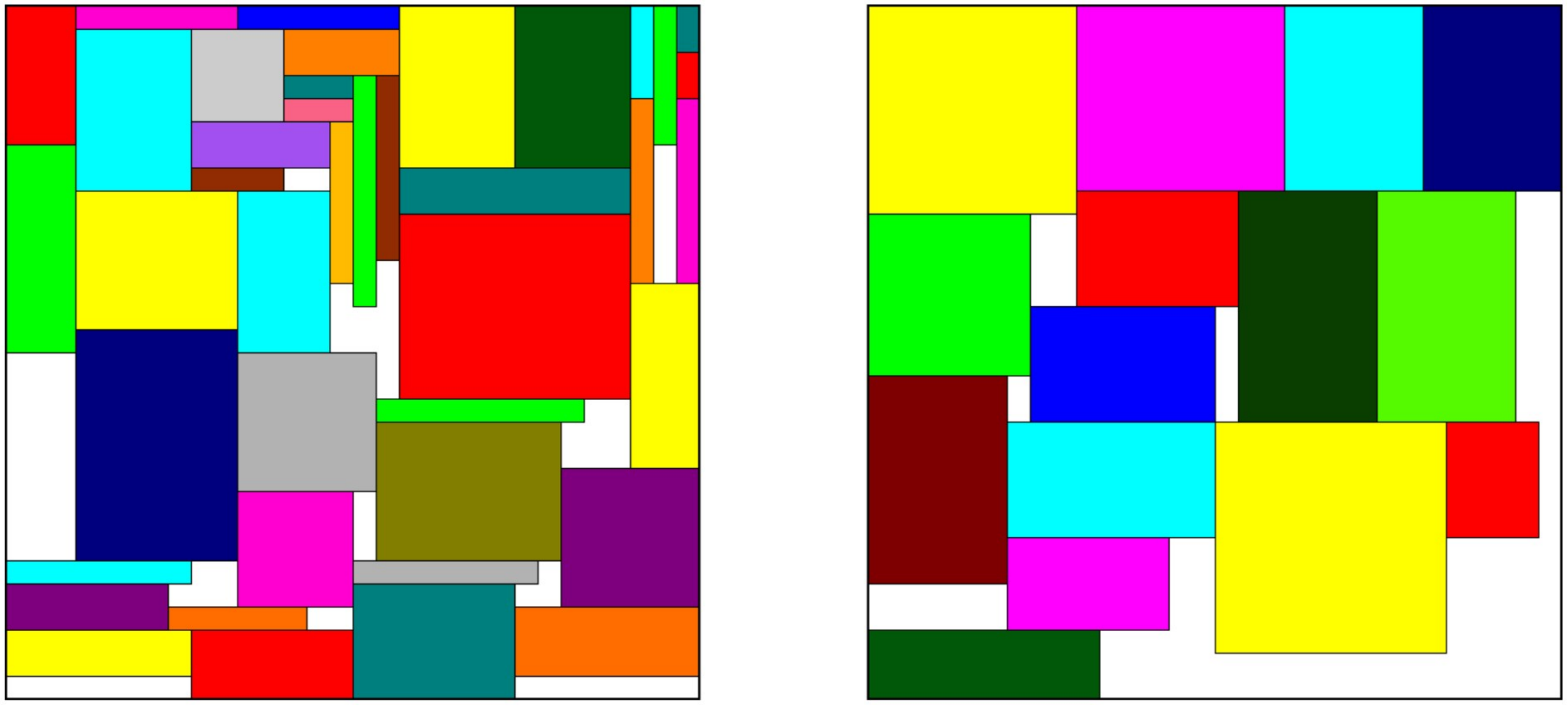
Example for 2D-BPP. Optimal configuration for an instance from [4] with two bins and more than 50 items.

The two-dimensional bin packing problem (2D-BPP) consists of packing without overlap, a set *I* of two-dimensional rectangular items into the minimum number of two-dimensional rectangular bins [1–3]. All the bins are identical with width *W* and height *H*, and each item *i* ∈ *I* has a specific width *w*_*i*_ and height *h*_*i*_. In several works of the literature, the authors consider the demand of each item independently, i.e., items with the same size are seen as different elements. In this study, we group the items with the same size and compute the demand for each one, represented by *d*_*i*_ for *i* = 1, …, *n*. We assume that all input data are positive integers and that *w*_*i*_ ≤ *W* and *h*_*i*_ ≤ *H*, for *i* = 1, …, *n*. Fig 1 shows the optimal solution for an instance from [4], which has more than 50 items to be packed. The white spaces are left-overs.

The 2D-BPP is strongly NP-hard since it generalizes the one-dimensional bin packing problem [3, 4], and according to the typology for cutting and packing problems proposed by [5], it belongs to the class of 2D-Single Bin-Size Bin Packing Problems. This problem has great significance for many industrial applications where it is required to cut small rectangular items from larger rectangular sheets of raw material such as textiles, glass, steel, wood, or paper [3, 6].

Bin-packing problems are at the heart of many interesting applications. In [7], an energy management problem in multi-core partitioned architectures requires bin-packing algorithms. In [8], a bioinformatic problem where the sequencing alignment processes is computationally challenging, a distributed and parallel indexing and alignment framework requires a greedy approximation algorithm for solving the bin-packing problem. Another application is cloud computing, which is considered one of the most promising technologies to meet customer demand flexibly. In [9], the authors mention that researchers have focused mainly on energy consumption and that the virtual machine placement problem is usually solved by using bin-packing algorithms. Recent applications in the delineation of rectangular management zones in agricultural fields have led to some of the preliminary ideas of the methodology presented here [10–12].

Bin packing problems are similar to cutting stock problems [13]. Indeed, in the bin packing problems, the item set is strongly heterogeneous, that is, there are many types of elements with small demand for each one. In the worst case, all the items have different sizes and demands equal to one. For cutting stock problems, the item set is weakly heterogeneous, thus there are a few types of items with high demand for each one [5, 14]. Excellent surveys of packing and cutting problems are presented in [3, 13, 15, 16], where most of the literature works refer to the case where the items have an orientation constraint (rotations by 90° are not allowed). However, the more general case, where 90° rotations are allowed, has not been broadly studied [2, 17]. In this article, we consider both cases with and without rotations. In [18] For the case when the items can be rotated by 90° the development of exact methods has been almost null [19] In this work, we propose an exact methodology that takes into account a set of valid positions for each item inside of the bin instead of a pattern configuration. To the best of our knowledge, this one of the first methodologies that solve the rotation case of the 2D-BPP for small and medium size instances.

Our two-stage methodology implemented for the 2D-BPP is called *Positions and Covering* (P&C). Given an instance of 2D-BPP, we compute a set of valid positions that indicate the possible ways of packing an item into the bin. This *preprocessing* is the key-point for the P&C. Then, the total number of bins *K* is fixed to a lower bound, and the P&C uses a set-covering model to solve the decision version of the 2D-BPP: is there a non-overlapping packing of the *n* items into the *K* bins? If there is a feasible solution, then *K* is the optimal value for the 2D-BPP instance. Otherwise, *K* is increased by one, and the set-covering model is solved again. The P&C iterates with the different *K* values until finding the optimal solution.

Some studies seek optimal solutions as we do. The authors in [20] propose a Lagrangian relaxation to solve an integer linear programming (ILP) model based on a discrete representation of the geometric space. In [4] are introduced combinatorial lower bounds and present an exact branch-and-bound approach. The works of [21–23], define a graph describing the overlaps of the items in the container from their projection on each orthogonal axis.

Another paper that is close to ours is [24], where the author develops a Lagrangian relaxation of a binary integer programming formulation to obtain an exact solution for the two-dimensional non-guillotine cutting problem. A guillotine cut splits a block into two smaller ones, where the slice plane is parallel to one side of the initial block. In this paper, we do not consider the guillotine cut constraint. In [25], the author presents different algorithms based on dynamic programming for unconstrained two-dimensional guillotine cutting.

Some of the best algorithms in the literature to solve the 2D-BPP are heuristics. In [26], the authors present a two-stage heuristic method using column generation and Lagrangian methods for a set-covering model. A Guided Local Search (GLS) is proposed by [27]. It starts with a greedy heuristic to obtain an upper bound on the number of bins. Then, the algorithm iteratively decreases this number searching for a feasible packing of the boxes using GLS.

The authors in [17, 28] propose a heuristic procedure to obtain new lower and upper bounds for the 2D-BPP. In [29], the authors implemented a Tabu Search proposed by [30–32] for general multi-dimensional bin packing problems. A Greedy Randomized Adaptive Search Procedure was developed by [33]. The authors in [1, 2] present genetic algorithms for the 2D-BPP. To the best of our knowledge, these algorithms yield the best results so far in terms of quality. With the P&C methodology we are able to prove that many of the heuristic solutions obtained by these methods are optimal.

The rest of this paper is organized as follows. In Section *Materials and methods*, we present our two-stage methodology to give optimal solutions for the 2D-BPP. In particular, three families of valid inequalities are introduced to enhance the set-covering integer formulation of the P&C methodology. Also, we extend the P&C methodology for the case when rotations by 90° are allowed. In Section *Results*, we experimentally validate the P&C methodology on a set of instances of small and medium size that have been broadly used in the literature so we can compare P&C with the best algorithms known so far. Finally, in Section *Conclusions*, we make some final remarks.

## Materials and methods

### The *positions and covering* methodology for the 2D-BPP

**Fig 2:**
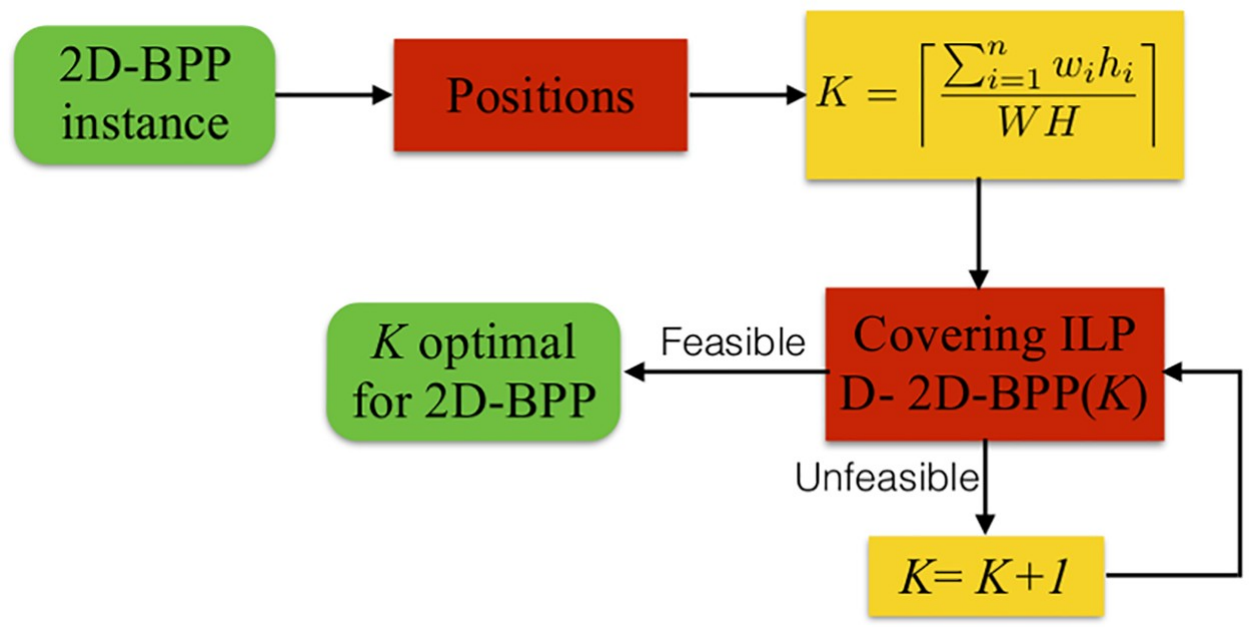
Scheme of the P&C. The two-step methodology to solve the 2D-BPP.

In Fig 2, we describe the *Positions and Covering* methodology. Given an instance of the 2D-BPP, the P&C methodology establishes a set of *valid positions* where each item could be placed into the bin (we prove that the total number of positions is of pseudo-polynomial size). Then the total number of bins *K* is fixed with (1)

$$\begin{array}{c} K=\lceil \frac{\sum_{i=1}^{n}w_{i}h_{i}}{WH}\rceil , \end{array}$$ (1)

that corresponds to the total area of the items divided by the area of the bin, that is, we assume that the items are perfectly packed (although the P&C methodology can use the best lower bound for each instance). The P&C considers the decision version of the 2D-BPP that we name as D-2D-BPP(*K*): is there a non-overlapping packing of the *n* items into *K* bins? The P&C exactly solves a covering ILP for the D-2D-BPP(*K*). If the D-2D-BPP(*K*) is feasible, then *K* is the optimal value for 2D-BPP. Otherwise, *K* is increased by one, and the covering model is solved again. Notice that *K* ≤ *n*, since in the worst case, we need *n* bins to pack all the items.

Naturally, better bounds and other alternatives to explore the feasible set of the number of bins could improve the number of iterations of the P&C. Some papers related to this task are [17, 28, 30]. In the rest of this section, we describe in detail the different stages of the P&C methodology for the 2D-BPP.

#### The *positions* stage of the P&C

The main idea of the *Positions* stage is to generate the dominant set of *valid positions* where the P&C can place an item into the bin. Indeed, from the infinite set of all the positions that an item can take in the bin, we only construct a finite set that guarantees the optimality of the solution. Notice that we are not enumerating or forming different patterns of items inside of the bin. Moreover, the *Position* stage must be only computed once in the P&C methodology.

**Fig 3:**
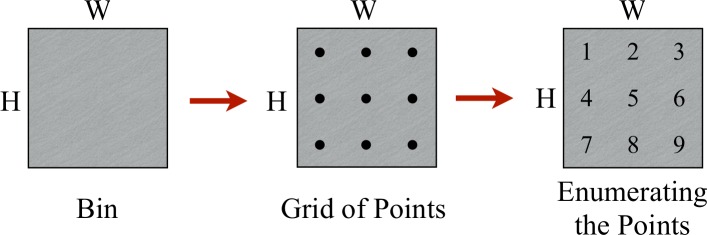
Grid inside of the bin. First step of the P&C methodology.

Let us consider a single bin with dimensions *H* × *W*. The first step in P&C is to delineate a Cartesian grid, that is, a regular tessellation of the 2-dimensional Euclidean space by congruent unit squares where each one represents a specific point that is enumerated and labeled. Fig 3 shows a bin with a size of 3 × 3 units.

**Fig 4:**
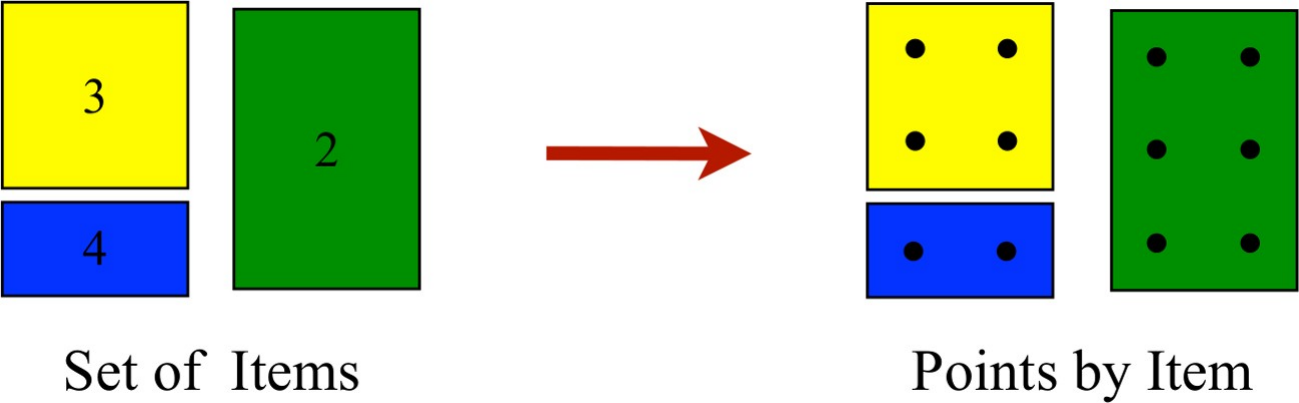
Points by item. The P&C methodology computes the points required for each item considering the grid inside of the bin.

Then, the P&C determines the number of points for each item considering its dimensions *h*_*i*_ × *w*_*i*_. Fig 4 shows the configuration for three items with sizes of 2 × 2 (yellow), 3 × 2 (green), and 1×2 (blue). The number inside of each item represents its demand.

With the pseudo-polynomial Algorithm 1 adapted to the 2D-BPP from [12], the P&C determines the set of valid positions for each item inside of the bin. The algorithm starts creating the positions of item *i* considering the width of the bin. After, it checks the height. The procedure is executed once for each item *i* = 1, …, *n*. The computational time required by Algorithm 1 depends on the size of the instance: the size of the bin and the total of items (see Theorem 1).

**Algorithm 1** *Positions(i)*

1: INPUT: *W*, *H*, *w*_*i*_, *h*_*i*_

2: **for** *j* = *w*_*i*_ **to** *W* **do**

3:  **for** *l* = 0 **to** (*W* − 1) **do**

4:   **if** (*j* + *l*) ≤ *W* **then**

5:    **for** *i* = *h*_*i*_ **to** *H* **do**

6:     **for** *k* = 0 **to** (*H* − 1) **do**

7:      **if** (*k* + *i*) ≤ *H* **then**

8:       creation and labeling of a new position for item *i*

9:      **end if**

10:     **end for**

11:    **end for**

12:   **end if**

13:  **end for**

14: **end for**

**Fig 5:**
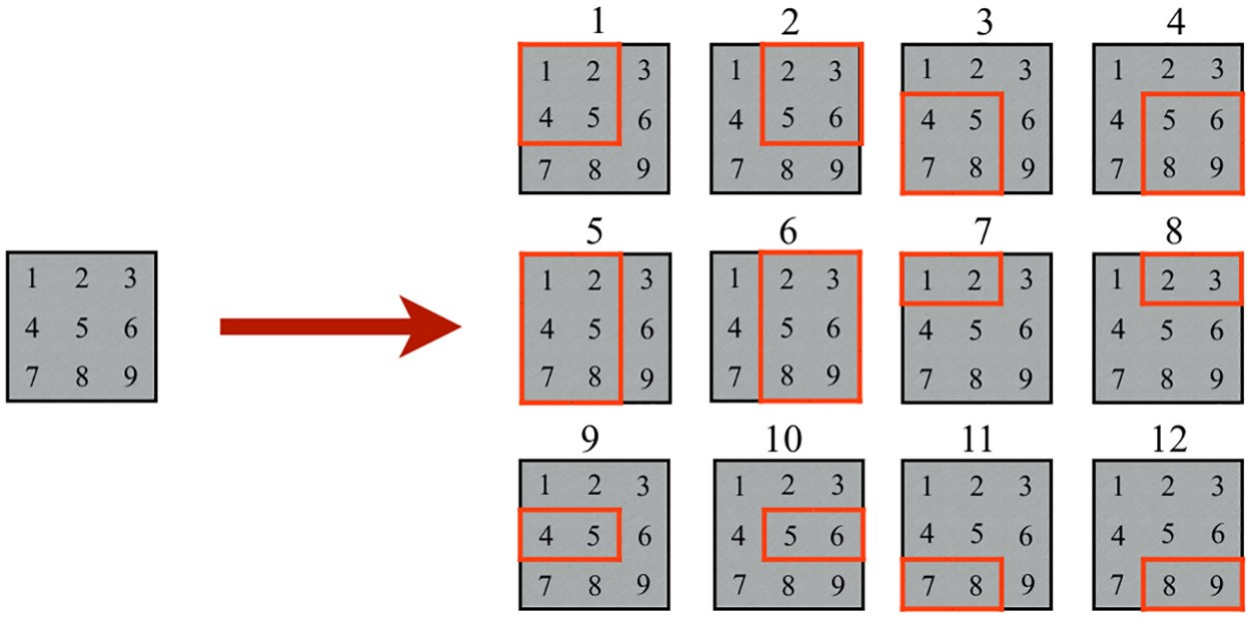
P&C without rotation. Set of valid positions when the 90° rotations are not allowed.

Fig 5 presents the set of valid positions for the bin and items of Figs 3 and 4. Notice that the rotation by 90 is not allowed for this instance, and each position has a specific label. Thus, positions 1-4 correspond to the first item, 5-6 to the second one, and 7-12 to the third one. By the integrality of the data, the set of valid positions is sufficient to obtain the optimal solution.

The results of Algorithm 1 can be mapped into a correspondence matrix *C* = {*c*_*jp*_} where rows represent the valid positions *J* of all the items and columns are the points *P* in the bin. Matrix *C* is composed of 1’s and 0’s, where *c*_*jp*_ = 1 if position *j* ∈ *J* includes point *p* ∈ *P*, and *c*_*jp*_ = 0 otherwise. Also, we obtain the subset of valid positions for each item *i* ∈ *I* called *T*(*i*) = {*j*|*γ*(*j*) = *i*} where function *γ*(*j*) indicates if position *j* is a valid position for item *i*, *T*(*i*) ⊂ *J*. The correspondence matrix of Fig 5 appears in Table 1.

**Table 1:** Correspondence matrix *C* for items of Fig 5.

	Position (*j*)	Point (*p*)								
		1	2	3	4	5	6	7	8	9
*Set of Valid Positions*	1	1	1	0	1	1	0	0	0	0
	2	0	1	1	0	1	1	0	0	0
	3	0	0	0	1	1	0	1	1	0
	4	0	0	0	0	1	1	0	1	1
	5	1	1	0	1	1	0	1	1	0
	6	0	1	1	0	1	1	0	1	1
	7	1	1	0	0	0	0	0	0	0
	8	0	1	1	0	0	0	0	0	0
	9	0	0	0	1	1	0	0	0	0
	10	0	0	0	0	1	1	0	0	0
	11	0	0	0	0	0	0	1	1	0
	12	0	0	0	0	0	0	0	1	1

Each row represents the respective position of Fig 5, e.g., row 1 has ones in points 1, 2, 4, and 5, that correspond to the same values as its label in the figure. The total number of valid positions for a set of items is pseudo-polynomial, as stated by Theorem 1.

**Theorem 1**. *The number of different valid positions that all the items in I can take in the Cartesian grid of a bin with size H* × *W is bounded by*

$$\begin{array}{c} \frac{W(W+1)H(H+1)}{2}. \end{array}$$

*Proof*. Recall that all input data are integers. Since there are *n* different items, we have that the number of different positions in a bin is

$$\begin{array}{c} \sum_{i=1}^{n}\left(W-w_{i}+1\right)\left(H-h_{i}+1\right). \end{array}$$

In the worst case, we have all possible different sizes of widths and heights, that is,

$$\begin{array}{c} \sum_{w=1}^{W}\sum_{h=1}^{H}H(W-w+1)(H-h+1)\\ \;=(W^{2}-\frac{W(W+1)}{2}+W)(H^{2}-\frac{H(H+1)}{2}+H)\\ \;=(\frac{W(W+1)}{2})(\frac{H(H+1)}{2}). \end{array}$$

Note that we use the partial sum of the first integers given by the triangular number.

#### The *covering* stage of the P&C

Now that we have the correspondence matrix *C* obtained by the *Positions* stage, we aim to find a feasible covering of the different positions of the items in the *K* bins, that is, we aim to solve the decision problem D-2D-BPP(*K*).

The Set-Covering formulation for the 2D-BPP has the following features. As mentioned, *I* is the set of items, and *J* is the set of valid positions of these items. Let *K* be the set of available bins, and *P* be the set of points into the bins. The parameters of the model are the correspondence matrix *C* = {*c*_*jp*_}, the subset of valid positions *j* for each item *i* represented by *T*(*i*), and the demand for each item *d*_*i*_.

The decision variables for the mathematical formulation are:

$$\begin{array}{c} x_{jk}^{i}=\{\begin{array}{cc} 1\; & \text{if}\;\text{the}\;\text{valid}\;\text{position}\;j\in T(i)\;\text{of}\;\text{item}\;i\in I\;\text{in}\;\text{bin}\;k\in K\;\text{is}\;\text{chosen,}\;\\ 0\; & \text{otherwise.}\; \end{array} \end{array}$$

The following covering model solves the decision problem D-2D-BPP(*K*):

minz=0 (2)

$$\begin{array}{ccc} \text{s.t.}\; & \\ & \sum_{i\in I}\sum_{j\in T(i)}c_{jp}x_{jk}^{i}\leq 1 & p\in P,k\in K, \end{array}$$ (3)

$$\begin{array}{ccc} & \sum_{j\in T(i)}\sum_{k\in K}x_{jk}^{i}\geq d_{i} & i\in I, \end{array}$$ (4)

$$\begin{array}{ccc} & x_{jk}^{i}\in \left\{0,1\right\} & i\in I,j\in T(i),k\in K. \end{array}$$ (5)

Notice, we do not force any objective function. Indeed in (2) any feasible solution that places all the items in the *K* bins is desirable. The restrictions (3) ensure that each point is covered by only one item for each bin. With this, P&C avoids the overlapping. The constraints (4) ensure that the demand for each item *i* is satisfied. Finally, in (5), the binary nature of the variables is presented.

If there is no a feasible solution for the previous covering model, then the P&C increases *K* by one and solves the model again until finding a feasible one, which will be the optimal solution, that is, there will exist enough bins to pack all the items in *I*.

#### Valid inequalities for the *covering* stage of the P&C

A valid inequality is a linear restriction that can be added to an ILP to make the polyhedron of the solution space to be closer to the convex hull of the discrete feasible solutions of the problem. This manner, fractional solutions may be avoided, and the computational time of solution methods based on the branch-and-bound algorithm can be reduced [34]. In the following, we present three families of valid inequalities for our set-covering mathematical formulation.

*Packing at least once each item* In the D-2D-BPP(*K*) model, we impose that the demand for the items must be satisfied (restrictions (4)). Let us now add a less restrictive condition, that is, each item must be at least once in the available bins:

$$\begin{array}{cc} \sum_{j\in T(i)}\sum_{k\in K}x_{jk}^{i}\geq 1\;\; & i\in I. \end{array}$$ (6)

Inequalities (6) are clearly valid by definition, and even if they are dominated by (4), the integer linear solvers are benefited from them (see Section Results).

*Packing at most M times each item into the bin* For each item *i*, it is possible to compute the maximum number of times, $M_{k}^{i}$, that can be packed into the bin *k*. Then the following inequality can be stated:

$$\begin{array}{cc} \sum_{j\in T(i)}x_{jk}^{i}\leq M_{k}^{i}\; & i\in I,k\in K. \end{array}$$ (7)

These inequalities are also valid by definition since they impose an upper bound for each item in each bin.

*Do not exceed the area of the bin* The total area of all the selected positions must not exceed the area of the bin. With this consideration, we obtain:

$$\begin{array}{cc} \sum_{i\in I}\sum_{j\in T(i)}\sum_{p\in P}c_{jp}x_{jk}^{i}\leq WH & \;k\in K. \end{array}$$ (8)

The mathematical formulation and the valid inequalities we propose are different from other approaches presented in the literature. Although these inequalities are simple to deduce, the experimental results show that they reduce, in a significant way, the computational time for some instances of the initial set-covering model.

### Rotation of the items

In the 2D-BPP, the rotation of the items by 90° is not allowed, that is, an item of size 3 × 2 is different from one of 2 × 3. However, in many applications, these two items represent the same one. In this section, we show that the P&C methodology can be adapted to the case when rotations are allowed.

First, the *Positions* stage must be extended to generate new rotated positions. Recall *T*(*i*) = {*j*|*γ*(*j*) = *i*} is a subset which indicates the valid positions *j* of item *i*. In this way, Algorithm 1 can be easily adapted. Notice that the incidence matrix *C* can almost double the number of its columns. Let $\bar{c_{jp}}$ correspond to the new columns of matrix *C* where $\bar{T(i)}=\{j|\gamma (j)=i\}$ is the subset of new valid positions *j* for item *i* with 90 rotations.

**Fig 6:**
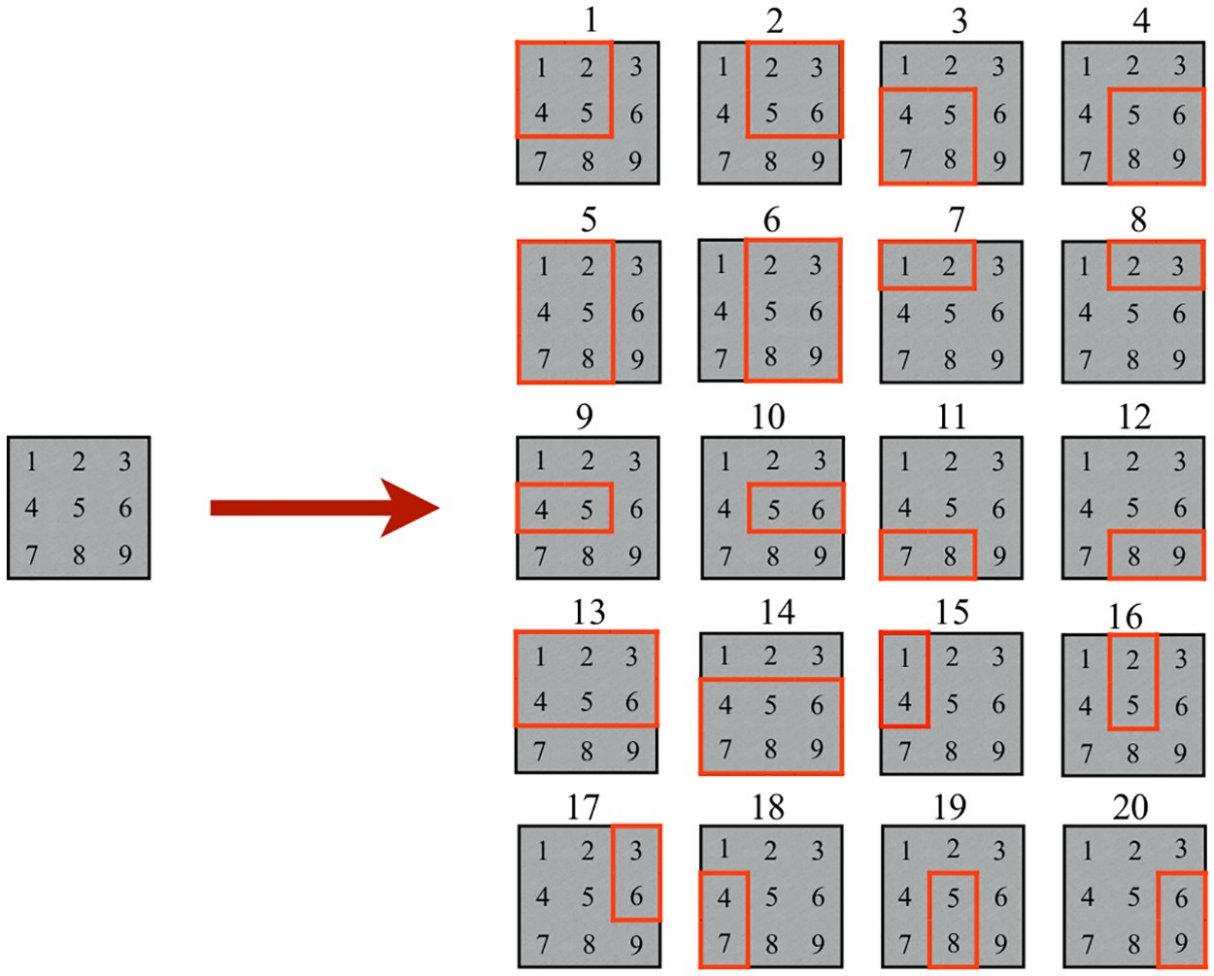
P&C with rotations. The new set of valid positions when rotations by 90° are allowed.

Fig 6 shows the new set of valid positions for the bin of size 3 × 3 and the set of items with sizes 2 × 2, 3 × 2, and 1 × 2. The new positions are labeled from 13 to 20. Notice that for the 2 × 2 item, Algorithm 1 has not created new valid positions.

The Set-Covering formulation must also be adapted using the following variables:

$$\begin{array}{c} y_{jk}^{i}=\{\begin{array}{cc} 1\; & \text{if}\;\text{the}\;\text{valid}\;\text{position}\;j\in \bar{T(i)}\;\text{of}\;\text{item}\;i\in I\;\text{in}\;\text{bin}\;k\in K\;\text{is}\;\text{chosen,}\;\\ 0\; & \text{otherwise.}\; \end{array} \end{array}$$

The following covering ILP solves the decision problem D-2D-BPP(*K*) with rotations:

minz=0 (9)

$$\begin{array}{ccc} \text{s.t.}\; & \\ & \sum_{i\in I}\sum_{j\in T(i)}c_{jp}x_{jk}^{i}+\sum_{i\in I}\sum_{j\in \bar{T(i)}}\bar{c_{jp}}y_{jk}^{i}\leq 1 & p\in P,k\in K, \end{array}$$ (10)

$$\begin{array}{ccc} & \sum_{j\in T(i)}\sum_{k\in K}x_{jk}^{i}+\sum_{j\in \bar{T(i)}}\sum_{k\in K}y_{jk}^{i}\geq d_{i} & i\in I, \end{array}$$ (11)

$$\begin{array}{ccc} & \sum_{j\in T(i)}\sum_{k\in K}x_{jk}^{i}+\sum_{j\in \bar{T(i)}}\sum_{k\in K}y_{jk}^{i}\geq 1 & i\in I, \end{array}$$ (12)

$$\begin{array}{ccc} & \sum_{j\in T(i)}x_{jk}^{i}+\sum_{j\in \bar{T(i)}}y_{jk}^{i}\leq M_{ik}\; & i\in I,k\in K, \end{array}$$ (13)

$$\begin{array}{ccc} & \sum_{i\in I}\sum_{j\in T(i)}\sum_{p\in P}c_{jp}x_{jk}^{i}\\ & \;+\sum_{i\in I}\sum_{j\in \bar{T(i)}}\sum_{p\in P}\bar{c_{jp}}y_{jk}^{i}\leq W\cdot H & \;k\in K, \end{array}$$ (14)

$$\begin{array}{ccc} & x_{jk}^{i}\in \left\{0,1\right\} & i\in I,j\in T(i),k\in K, \end{array}$$ (15)

$$\begin{array}{ccc} & y_{jk}^{i}\in \left\{0,1\right\} & i\in I,j\in \bar{T(i)},k\in K. \end{array}$$ (16)

As in the non-rotation case, P&C only seeks for feasibility with objective function (9). The restrictions (10) ensure that each point of each bin is covered by no more than one item, whether rotated or not. Constraints (11) ensure that the demand for each item *i* is satisfied by the rotated and non-rotated positions. Constraints (12)–(14) correspond to the valid inequalities (6)–(8). Finally, the binary nature of variables is represented by (15) and (16).

If there is no feasible solution for the covering model, then P&C increases the number of bins *K* by one and solves the model again until finding a feasible one, which corresponds to the optimal solution.

## Results

In this section, we present the experimental results to validate the P&C efficiency, considering the classical benchmark instances for the 2D-BPP. First, we show experimental results of the set-covering ILP together with different combinations of the valid inequalities we have proposed. Then, we make a comparison with the metaheuristic results presented in [2] and with two exact methodologies presented by [4] and [35].

Table 2 summarizes the instance benchmark we used to solve the 2D-BPP with the P&C methodology together with a short description for each class. The *bwmv* instances and their corresponding averages of the best-known values, are available at http://or.dei.unibo.it/library/two-dimensional-bin-packing-problem. The *beng* instances are available in the PackLib2 library, which can be downloaded from http://www.ibr.cs.tu-bs.de/alg/packlib/index.shtml. These two sets of instances are specially conceived for the 2D-BPP. The *cgcut* and the *ngcut* instances were initially conceived for two-dimensional cutting problems and were transformed into 2D-BPP instances accordingly to [4]. This set of instances can be downloaded from the OR-library available at http://people.brunel.ac.uk/~mastjjb/jeb/info.html.

**Table 2:** Benchmark instances for the 2D-BPP.

Instance Class	Description
*bwmv*:	500 instances proposed by [4] and [36] for the 2D-BPP, divided into ten classes: each class comprises 50 instances, 10 for each number of items *n* ∈ {20, 40, 60, 80, 100}.
*cgcut*:	3 instances proposed by [20].
*ngcut*:	12 instances proposed by [24].
*beng*:	10 instances proposed by [37] for the 2D-BPP.

For the P&C algorithm, the *Positions* stage was coded in C++ and executed on a MAC Pro equipped with an Intel Core i7 processor of 2 GHz and 8 GB of RAM. To solve the set-covering model, coded in C++ using Concert Technology, we used the integer linear solver (B&B) of CPLEX 12.7 fixing the gap at 0% and the time limit to 5 hours. The B&B was executed on a computer equipped with 64 GB of RAM, and a processor 8-Core Intel Xeon E5-2609 @1.70 GHz.

### The *positions* stage of the P&C methodology

The execution time required to obtain the set of valid positions for each class of instances by using Algorithm 1 at the *Positions* stage of the P&C methodology is presented in Tables 3 and 4. Table 3 presents the instances for Classes 1–3 of the *bwmv* instances with and without 90° rotations, showing the class in the first column, the size of the bin in the second one, the number of items in the third, the time in seconds for the non-rotation case in the fifth, and the time in seconds for the rotation case in the last column. The resulting time is the average of ten instances.

**Table 3:** Execution time for the *Positions* stage of P&C, Algorithm 1, for the instances in Classes 1–3 of *bwmv* with and without rotations.

Class	W ×	*n*	Non-rotation time (sec)	Rotation time (sec)
1	10×10	20	0.008	0.017
		40	0.015	0.034
		60	0.021	0.044
		80	0.025	0.050
		100	0.030	0.059
2	30×30	20	1.537	2.829
		40	2.498	4.953
		60	3.329	6.727
		80	4.290	8.191
		100	5.207	9.222
3	40×40	20	2.369	4.359
		40	4.591	9.513
		60	6.923	13.920
		80	8.869	17.588
		100	10.512	22.516

**Table 4:** Execution time for the *Positions* stage of P&C, Algorithm 1, for the *cgcut*, *ngcut*, and *beng* instances with and without rotations.

	Class	*W* × *H*	*n*	Non-rotation time (sec)	Rotation time (sec)
*cgcut*	1	15×10	16	0.014	0.024
	2	40×70	23	5.372	10.920
	3		62	5.617	10.700
*ngcut*	1	10×10	10	0.002	0.004
	2		17	0.003	0.007
	3		21	0.006	0.013
	4	15×10	7	0.005	0.008
	5		14	0.006	0.008
	6		15	0.013	0.031
	7	20×20	8	0.042	0.089
	8		13	0.059	0.120
	9		18	0.082	0.166
	10	30×30	13	0.060	0.115
	11		15	0.262	0.528
	12		22	0.311	0.701
*beng*	1	25×30	20	0.888	1.824
	2	25×10	40	0.136	0.215
	3		60	0.202	0.316
	4		80	0.250	0.506
	5		100	0.286	0.477
	6	40×25	40	3.164	5.554
	7		80	5.844	10.465
	8		120	6.999	12.241
	9		160	7.720	13.872
	10		200	8.687	15.383

From Table 3, we point out that for the instances in Classes 1–3, the time consumed at the *Positions* stage is not relevant as it does not reach more than 11 seconds for the non-rotation case and 23 seconds for the rotation case considering the harder instances. Nevertheless, this stage is pseudo-polynomial, so it grows with the size of the bin and the number of items. Indeed, we tested Algorithm 1 for the non-rotation case with the instances in the Classes 4–5 and 7–10 that consider bins with a size of 100 × 100. The minimum computational time required to obtain the set of valid positions was 112 seconds and the maximum was 884. For Class 6, where bins with a size of 300 × 300 are considered, it was not possible to obtain the set of valid positions in less than two hours (see [4] and [36] for more details about these instance classes).

Table 4 is similar to the previous one. It shows the execution time required at the *Positions* stage of the P&C methodology, by using Algorithm 1, for the *cgcut*, *ngcut*, and *beng* instances with and without rotations by 90°. The first column presents the class, the number of instance is in the second column, the size of the bin is in the third one, the number of items in the fourth one, the time in seconds for the non-rotation case in the fifth column, and finally, the time in seconds for the rotation case is in the last column. For this table, the resulting time is for each instance.

From Table 4 we can again observe that the time required by Algorithm 1 to generate the possible positions that the items can take into the bin is not time-consuming as no more than 9 seconds are needed for the non-rotation case and 16 seconds for the rotation case. Nevertheless, the time will increase as the instances get larger. Notice that for the rotation case the times can almost double the original time, but it remains reasonable.

### P&C: Experimental results

The P&C methodology starts with the determination of all the possible positions where an item can be placed, and then, in the *Covering* stage, a set-covering ILP is executed to validate if the items can be placed in K bins. In this section, we validate the efficiency of the proposed sets of valid inequalities to strengthen the initial set-covering ILP of the *Covering* stage of the P&C methodology.

Tables 5–7 show the results for the *bwmv* instances (Classes 1, 2, and 3, respectively), without rotation of the items. Class 1 considers bins with a size of 10 × 10, Class 2 bins of 30 × 30, and Class 3 bins of 40 × 40. For each table, the first column indicates the number of the instance, and the second one is the optimal solution obtained by P&C, which corresponds to the number of bins required to pack all the items. Columns 3 to 10 show the execution time (*seconds*) required by the initial set-covering ILP model (Original Model, without valid inequalities) and the ILP model with different incorporations of valid inequalities. This manner, the fourth column considers the set-covering ILP with the addition of valid inequality (6), and the last column considers the incorporation of the three families (6), (7), and (8). “*T.O.*” means the execution time limit was reached, and it was not enough to find a solution (*Time Over*). Recall that the set-covering ILP is a decision problem; thus, we cannot obtain information about the proximity with the optimal solution, only a feasible or unfeasible answer. Numbers in bold represent the best times.

**Table 5:** Experimental results for Class 1 of the *bwmv* instances without rotations, applying the different families of valid inequalities to the set-covering ILP.

Instance	Solution	Execution time *(seconds)*							
		Original Model	Valid(s) Inequality(s)						
			(6)	(7)	(8)	(6) & (7)	(6) & (8)	(7) & (8)	(6)–(8)
1	8	**0.5**	**0.5**	**0.5**	**0.5**	**0.5**	0.6	**0.5**	**0.5**
2	5	0.6	0.6	0.7	**0.5**	**0.5**	**0.5**	**0.5**	**0.5**
3	9	**0.0**	0.7	**0.0**	**0.0**	0.7	0.8	**0.0**	0.8
4	6	**0.5**	**0.5**	**0.5**	**0.5**	**0.5**	0.6	**0.5**	0.6
5	6	**0.1**	**0.1**	0.2	0.5	**0.1**	0.6	0.5	0.6
6	9	1.7	**0.7**	1.7	6.4	**0.7**	1.0	6.4	1.1
7	6	0.6	0.6	0.6	0.6	**0.5**	0.6	0.7	0.6
8	6	0.7	**0.6**	0.7	0.7	0.8	0.9	0.7	0.9
9	8	**0.0**	0.7	**0.0**	**0.0**	0.8	0.7	**0.0**	0.7
10	8	0.7	0.8	0.7	0.7	**0.6**	1.9	0.7	**0.6**
11	10	32.5	21.1	32.7	1.9	41.5	**1.5**	2.0	1.6
12	12	2.2	**1.9**	2.3	2.3	**1.9**	2.2	2.3	2.2
13	17	**2.3**	**2.3**	2.4	3.5	2.4	2.5	3.5	2.5
14	14	4.7	16.1	4.9	13.0	**2.3**	5.7	13.0	13.0
15	15	**3.7**	43.1	4.1	24.3	4.2	39.5	24.4	12.5
16	14	2.2	**1.9**	2.7	2.1	2.0	2.3	2.2	2.1
17	11	59.7	66.1	60.0	**35.9**	65.7	57.1	**35.9**	57.1
18	19	**0.0**	2.3	**0.0**	**0.0**	2.3	2.4	**0.0**	2.5
19	11	4.2	**2.1**	4.4	3.5	2.2	35.4	3.5	35.3
20	11	31.1	39.6	31.6	**9.6**	39.7	28.0	**9.6**	28.0
21	23	8.1	9.2	8.4	**8.0**	9.4	47.3	8.2	47.3
22	19	50.7	42.7	51.1	45.2	43.3	**29.4**	45.2	**29.4**
23	21	**21.4**	45.1	21.6	41.3	45.6	23.2	41.4	23.3
24	22	**8.1**	28.0	8.4	29.3	27.8	30.6	29.4	30.6
25	19	6.6	4.7	6.9	7.0	4.7	**3.3**	7.3	3.4
26	17	1300.4	257.3	1294.4	723.7	**255.6**	269.8	725.9	271.4
27	16	52.9	79.7	53.1	**5.3**	79.9	6.3	**5.3**	6.3
28	21	**27.9**	74.1	28.3	64.4	35.8	35.4	65.0	51.8
29	18	239.4	145.9	240.8	**126.1**	184.3	185.8	126.4	162.7
30	24	16.1	**7.1**	16.4	28.3	7.2	37.5	28.3	37.6
31	25	139.9	98.5	127.5	94.1	173.1	**51.4**	64.2	78.6
32	26	66.4	232.8	222.6	148.6	75.8	**60.7**	76.0	72.8
33	27	80.4	47.2	**44.8**	73.0	149.6	106.1	121.2	52.3
34	27	79.4	62.7	69.0	113.4	75.6	71.1	85.5	**45.2**
35	26	119.2	198.6	119.3	65.0	198.3	**44.2**	65.3	44.4
36	28	107.8	152.0	108.7	**88.3**	153.1	118.3	88.7	118.3
37	31	**44.6**	52.7	44.9	48.0	52.9	49.5	47.9	49.5
38	29	114.0	59.5	121.0	64.0	81.0	111.0	**58.3**	129.1
39	30	76.8	358.4	73.6	260.8	107.7	**66.5**	965.8	332.1
40	26	183.7	133.9	270.8	71.4	**53.0**	311.6	119.6	88.6
41	28	363.7	660.3	364.5	1334.3	875.9	982.4	**306.0**	445.0
42	31	1181.8	1504.7	1177.3	1069.2	1503.5	1183.5	**646.8**	1956.0
43	29	110.6	88.5	137.4	80.1	145.1	**63.3**	133.8	174.9
44	30	1702.7	866.2	1704.0	614.4	864.2	748.6	447.1	**288.5**
45	32	457.0	531.8	454.6	233.3	531.1	**90.8**	230.9	603.6
46	37	169.4	261.9	252.2	127.8	**91.3**	142.1	101.7	575.1
47	28	709.4	836.1	712.2	766.2	1012.4	819.2	**290.8**	642.7
48	33	1230.0	1943.7	736.5	1090.3	932.5	**301.9**	1014.5	1108.1
49	31	829.2	439.1	833.4	1285.2	**438.8**	628.6	572.6	639.0
50	38	611.7	829.9	608.3	227.0	829.9	268.6	**96.2**	130.6
**Total Time**		10257.0	10254.2	10062.3	9039.3	9207.9	7072.5	**6721.9**	8401.5

**Table 6:** Experimental results for Class 2 of the *bwmv* instances without rotations, applying the different families of valid inequalities to the set-covering ILP.

Instance	Solution	Execution time *(seconds)*							
		Original Model	Valid(s) Inequality(s)						
			(6)	(7)	(8)	(6) & (7)	(6) & (8)	(7) & (8)	(6)–(8)
1	1	**0.0**	9.9	**0.0**	**0.0**	10.1	10.2	**0.0**	10.3
2	1	**0.0**	3.7	**0.0**	**0.0**	3.8	4.9	**0.0**	4.9
3	1	**0.0**	12.7	**0.0**	**0.0**	12.8	13.1	**0.0**	13.1
4	1	**0.0**	6.1	**0.0**	**0.0**	6.1	6.4	**0.0**	6.4
5	1	**0.0**	5.3	**0.0**	**0.0**	5.3	6.9	**0.0**	7.0
6	1	**0.0**	0.2	**0.0**	**0.0**	0.2	0.2	**0.0**	0.2
7	1	**0.0**	6.8	**0.0**	**0.0**	7.0	7.1	**0.0**	7.2
8	1	**0.0**	6.7	**0.0**	**0.0**	6.8	6.9	**0.0**	7.0
9	1	**0.0**	10.4	**0.0**	**0.0**	10.5	10.7	**0.0**	10.8
10	1	**0.0**	12.1	**0.0**	**0.0**	12.1	12.2	**0.0**	12.3
11	1	0.1	13.1	**0.0**	0.1	13.5	15.9	0.1	16.1
12	2	**0.1**	25.3	**0.1**	**0.1**	25.5	26.1	**0.1**	26.3
13	2	**0.1**	37.8	**0.1**	**0.1**	38.1	38.9	**0.1**	39.1
14	2	**0.1**	35.3	**0.1**	**0.1**	35.4	36.0	**0.1**	36.4
15	2	**0.1**	37.3	**0.1**	**0.1**	37.6	38.2	**0.1**	38.5
16	2	**0.1**	28.1	**0.1**	**0.1**	28.0	28.8	**0.1**	29.0
17	2	**0.1**	18.8	**0.1**	**0.1**	19.0	19.1	**0.1**	19.2
18	2	**0.1**	48.6	**0.1**	**0.1**	49.3	50.4	**0.1**	50.3
19	2	**0.1**	20.3	**0.1**	**0.1**	20.7	21.1	**0.1**	21.4
20	2	**0.1**	15.6	**0.1**	**0.1**	15.8	18.7	**0.1**	18.9
21	3	**0.2**	65.5	**0.2**	**0.2**	66.2	67.0	**0.2**	67.6
22	2	**0.1**	52.3	0.2	0.2	52.9	53.9	0.2	54.4
23	3	**0.2**	58.5	**0.2**	**0.2**	59.2	59.8	**0.2**	60.2
24	3	**0.2**	72.0	**0.2**	**0.2**	72.7	74.0	**0.2**	74.9
25	2	**0.1**	51.8	0.2	**0.1**	52.3	53.2	**0.1**	53.8
26	2	**0.1**	44.9	**0.1**	**0.1**	45.6	46.4	**0.1**	47.0
27	2	**0.1**	34.2	**0.1**	**0.1**	35.1	42.7	**0.1**	43.1
28	3	**0.2**	68.8	**0.2**	**0.2**	69.4	70.8	**0.2**	71.5
29	2	0.2	50.5	0.2	**0.1**	51.2	51.9	0.2	52.8
30	3	**0.2**	84.7	**0.2**	**0.2**	85.6	87.5	**0.2**	87.7
31	3	**0.3**	85.0	**0.3**	**0.3**	86.4	87.7	**0.3**	88.8
32	3	**0.3**	100.1	**0.3**	**0.3**	101.2	100.8	**0.3**	102.7
33	3	**0.3**	102.0	**0.3**	**0.3**	103.2	104.7	**0.3**	106.1
34	3	**0.3**	102.2	**0.3**	**0.3**	103.4	105.0	**0.3**	107.3
35	3	**0.2**	76.9	0.3	**0.2**	77.9	93.8	0.3	95.7
36	3	**0.3**	101.0	**0.3**	**0.3**	102.1	104.8	**0.3**	105.1
37	3	**0.3**	119.4	**0.3**	**0.3**	121.6	232.4	**0.3**	234.0
38	3	**0.3**	108.8	**0.3**	**0.3**	110.3	209.4	**0.3**	210.4
39	4	**0.3**	235.5	**0.3**	**0.3**	235.4	255.4	**0.3**	258.9
40	3	**0.3**	102.8	**0.3**	**0.3**	104.7	105.2	**0.3**	106.3
41	3	**0.3**	92.9	**0.3**	**0.3**	94.7	210.1	**0.3**	212.2
42	4	**0.4**	236.3	**0.4**	**0.4**	237.9	259.1	**0.4**	274.0
43	3	**0.3**	110.1	**0.3**	**0.3**	111.5	218.6	**0.3**	219.4
44	4	**0.4**	230.2	**0.4**	**0.4**	232.4	252.6	**0.4**	261.7
45	4	**0.4**	238.8	**0.4**	**0.4**	240.8	268.4	**0.4**	287.7
46	4	**0.4**	297.7	**0.4**	0.5	323.9	355.8	0.5	361.7
47	4	**0.3**	92.0	**0.3**	**0.3**	93.6	208.5	**0.3**	210.5
48	4	**0.4**	282.0	**0.4**	**0.4**	288.0	338.4	**0.4**	350.3
49	4	**0.4**	239.6	**0.4**	**0.4**	241.3	263.8	**0.4**	272.0
50	4	**0.4**	291.4	**0.4**	0.5	373.1	360.0	0.5	362.3
**Total Time**		**8.8**	4181.8	9.2	9.2	4330.9	5113.4	9.2	5214.1

**Table 7:** Experimental results for Class 3 of the *bwmv* instances without rotations with *n* = 20 and *n* = 40, applying the different families of valid inequalities to the set-covering ILP.

Instance	Solution	Execution time *(seconds)*							
		Original Model	Valid(s) Inequality(s)						
			(6)	(7)	(8)	(6) & (7)	(6) & (8)	(7) & (8)	(6)–(8)
1	6	124.6	**116.6**	124.9	127.1	117.8	122.1	127.0	122.9
2	3	10325.3	10323.1	*T.O.*	**86.3**	*T.O.*	86.7	188.8	188.4
3	6	**0.4**	119.2	**0.4**	**0.4**	119.8	120.9	**0.4**	121.7
4	4	71.3	**69.5**	71.0	74.4	69.9	219.1	73.8	69.9
5	4	1409.2	1424.8	11093.3	**90.9**	11155.9	92.6	1357.7	1359.2
6	7	**0.4**	0.6	**0.4**	**0.4**	0.6	0.6	**0.4**	0.6
7	5	**0.2**	67.8	**0.2**	**0.2**	68.1	69.6	**0.2**	69.1
8	4	151.8	136.7	129.4	108.9	147.5	133.4	105.3	**103.0**
9	5	**201.0**	202.8	222.8	529.4	223.9	527.7	1431.0	1432.6
10	7	**0.4**	**0.4**	**0.4**	**0.4**	**0.4**	**0.4**	**0.4**	**0.4**
11	—	*T.O.*	*T.O.*	*T.O.*	*T.O.*	*T.O.*	*T.O*	*T.O.*	*T.O.*
12	—	*T.O.*	*T.O.*	*T.O.*	*T.O.*	*T.O.*	*T.O.*	*T.O.*	*T.O.*
13	11	3219.7	**782.7**	*T.O.*	3329.5	3561.7	2464.9	4596.8	3721.2
14	10	1208.8	**620.9**	2752.0	1980.9	822.27	623.1	942.3	717.9
15	12	1.3	1.3	1.3	1.3	1.3	1.3	1.3	**1.2**
16	10	516.3	519.2	480.9	493.8	**342.3**	505.1	525.5	503.1
17	—	*T.O.*	*T.O.*	*T.O.*	*T.O.*	*T.O.*	*T.O.*	*T.O.*	*T.O.*
18	13	428.8	764.6	**441.0**	689.9	1235.4	733.2	793.8	734.1
19	8	**449.4**	450.4	451.8	442.8	2724.4	598.7	450.5	453.3
20	8	**489.1**	1094.2	872.3	533.9	932.1	1304.9	551.1	551.6
**Total Time**		72598.2	70694.8	106642.2	62490.7	93523.3	**61604.3**	65146.4	64150.3

Table 5 clearly shows that there is no better model than another to solve a specific class of instances. Even the original model without valid inequalities is, for some instances, advantageous in terms of time since it has fewer restrictions to handle by the branch-and-bound algorithm of CPLEX. The ILP model with the three valid inequalities has a most significant number of constraints, but they strengthen the convex hull of the solution polyhedra, since for some instances, this combination is better than the others. In terms of the total time required to solve all the instances of Class 1, we point out that the ILP enhanced with valid inequalities (7) & (8) is the less time-consuming.

Table 6 shows another aspect of our experimental results. Class 2 of the *bwmv* instances have bins of size 30 × 30. The instances seem more straightforward than the ones of Class 1 since the largest execution time is around six minutes. The original model without valid inequalities is, for most of the instances, advantageous in terms of time. Indeed, having fewer restrictions to handle along the branch-and-bound algorithm of CPLEX seems to be advantageous for these instances. Nevertheless, the ILP enhanced with valid inequalities (7) & (8) is still competitive.

Experimental results of Table 7 show that Class 3 has instances that are more time-consuming and more challenging than Class 2. Indeed, the bin size is 40 × 40, and our approach could solve only the instances with 20 and 40 items. As for Class 1, there is not a clear ILP that overruns all other models, but we can point out the set-covering ILP with inequalities (6) & (8) since it has the lowest total time to solve all the instances if we consider *T.O*. with a value of 18000 seconds (execution time).

The experimental results of the P&C methodology for the *cgcut*, *ngcut*, and *beng* instances are shown in Table 8. This table is similar to previous ones, but in this case, we add a new column (the first column) to indicate the class of the instance. The valid inequality with the best performance is (8) since it has the lowest total time and considering that it solved all the instances within the time-limit. For these instances, the original model is not competitive, so the importance of valid inequalities is again validated.

**Table 8:** Experimental results for *cgcut*, *ngcut*, and *beng* instances without rotations.

Class	Instance	Solution	Execution time *(seconds)*							
			Original Model	Valid(s) Inequality(s)						
				(6)	(7)	(8)	(6) & (7)	(6) & (8)	(7) & (8)	(6)–(8)
*cgcut*	1	2	**0.0**	0.2	**0.0**	**0.0**	0.2	0.1	**0.0**	0.1
	2	2	**0.1**	28.6	**0.1**	**0.1**	28.7	29.5	**0.1**	29.3
	3	23	**2334.8**	2367.4	2386.1	2340.5	2385.8	2375.8	2380.1	2391.0
*ngcut*	1	3	**0.0**	**0.0**	**0.0**	**0.0**	**0.0**	**0.0**	**0.0**	**0.0**
	2	4	**0.0**	0.2	**0.0**	**0.0**	0.2	0.2	**0.0**	0.2
	3	3	1.4	1.3	1.3	1.1	1.4	**0.3**	1.2	1.2
	4	2	**0.0**	**0.0**	**0.0**	**0.0**	**0.0**	**0.0**	**0.0**	**0.0**
	5	3	**0.0**	0.1	**0.0**	**0.0**	0.1	0.1	**0.0**	0.1
	6	3	**0.0**	0.3	**0.0**	**0.0**	0.3	0.3	**0.0**	0.3
	7	1	**0.0**	0.1	**0.0**	**0.0**	**0.0**	0.1	**0.0**	0.1
	8	2	**0.2**	**0.2**	**0.2**	**0.2**	**0.2**	**0.2**	**0.2**	**0.2**
	9	3	1.7	**1.6**	1.8	1.7	**1.6**	1.7	1.8	1.7
	10	3	**0.0**	1.9	**0.0**	**0.0**	1.9	2.0	**0.0**	1.9
	11	2	**3.2**	7.6	**3.2**	3.3	7.5	3.7	3.3	3.7
	12	3	5.9	**5.6**	5.9	6.0	**5.6**	5.7	6.0	5.7
*beng1-8*	1	4	**0.0**	4.4	**0.0**	**0.0**	4.4	4.5	**0.0**	4.5
	2	6	255.6	338.5	254.8	**222.9**	339.6	273.3	223.2	274.4
	3	9	571.5	629.3	609.7	728.4	1033.4	576.2	**494.7**	769.4
	4	11	5292.5	4588.5	6377.2	1571.2	2252.0	**630.0**	2615.6	2320.4
	5	14	4343.4	6806.4	3336.0	**1118.7**	4650.3	3430.1	2010.5	2333.6
	6	2	**0.0**	4.0	**0.0**	**0.0**	4.1	9.4	**0.0**	9.4
	7	3	9467.6	*T.O.*	*T.O.*	111.2	9134.4	93.4	111.6	**93.2**
	8	5	**0.2**	4211.8	**0.2**	**0.2**	*T.O.*	49.5	**0.2**	50.0
*beng9-10*	1	6	**0.3**	*T.O.*	**0.3**	**0.3**	*T.O.*	63.3	**0.3**	64.1
	2	7	**0.4**	*T.O.*	**0.4**	**0.4**	*T.O.*	82.0	**0.4**	82.5
**Total Time**			22278.7	72998.0	30977.1	**6106.3**	73851.4	7630.9	7849.1	8436.9

We compare the P&C methodology with the approaches that have proved to be the most effective in the literature. Table 9 gives a short description of each one. The first nine approaches correspond to metaheuristic methods, while the last two are exact ones. As it is mentioned in [19], many mathematical models and solution methods have been proposed for the bin packing problem. However, no exact mathematical models have been presented for the 2D-BPP with rotations of the items by 90°.

**Table 9:** Comparative approaches for the 2D-BPP.

Approach	Description
TS3:	Tabu search based on the constructive procedures of [30].
HBP:	Constructive heuristic that assigns a score to each item by [17].
GLS:	Guided local search heuristic based on the iterative solution of constraint satisfaction problems proposed by [27].
SCH:	Set-covering-based heuristic approach presented by [26].
GVND:	Hybrid GRASP/VND algorithm by [33].
BRKGA:	Biased random key genetic algorithm by [2]. This algorithm allows 90° rotation of the items.
HHA-NO(r):	Hyper-heuristic algorithm based on rotation mutation operators proposed by [38]. This algorithm allows rotation of the items.
HHA-NO(sr):	Hyper-heuristic algorithm based on swap-rotation mutation operators proposed by [38]. This algorithm allows rotation of the items.
SVC2BPRF:	A sequential value correction heuristic proposed by [39] allows rotation of the items.
BBMV:	Branch-and-bound algorithm presented by [4]
DCSP:	Decomposition technique with constraint programming by [35]

Tables 10 and 11 present a comparison of the results obtained by P&C against the approaches from Table 9. Table 10 shows the results for the *bwmv* instances. The first column indicates the class of the *bwmv* group, the second column the bin size *W* × *H*, and the third column the number of items to be packed into the bins. The fourth column is the lower bound reported by [26], computed by applying all the lower-bounding procedures from the literature and an exact algorithm for long computing time. The fifth column indicates the optimal number of bins obtained by the P&C methodology with valid inequalities (7) and (8). The rest of the columns indicate the solutions (number of bins) for the other approaches: BRKGA, GVND, SCH, GLS, TS3, HBP, BBMV, and DCSP, respectively. Each row corresponds to the average values of the number of bins for ten instances of each class-size. Entries with “*T.O.*” mean that the approach could not find a feasible solution within the time limit. Entries with *u*/*v* mean that only *u* instances out of *v* from this class are solved within the time limits of each approach. Notice that the experimental results of the approaches we are comparing with were done on different computers, so this table is more informative than comparative.

**Table 10:** Comparison of the solutions obtained by the different approaches for the *bwmv* instances without rotations.

Class	*W* × *H*	*n*	LB*	P&C	BRKGA	GVND	SCH	GLS	TS3	HBP	BBMV	DCSP
1	10×10	20	7.1	**7.1**	**7.1**	**7.1**	**7.1**	**7.1**	**7.1**	**7.1**	**7.1**	**7.1**
		40	13.4	**13.4**	**13.4**	**13.4**	**13.4**	**13.4**	13.5	**13.4**	**13.4**	**13.4**
		60	19.7	**20**	**20**	**20**	**20**	20.1	20.1	20.1	7/10	**20**
		80	27.4	**27.5**	**27.5**	**27.5**	**27.5**	**27.5**	28.2	**27.5**	3/10	**27.5**
		100	31.7	**31.7**	**31.7**	**31.7**	**31.7**	32.1	32.6	31.8	1/10	**31.7**
2	30×30	20	1.0	**1.0**	**1.0**	**1.0**	**1.0**	**1.0**	**1.0**	**1.0**	**1.0**	**1.0**
		40	1.9	**1.9**	**1.9**	**1.9**	**1.9**	**1.9**	2.0	**1.9**	**1.9**	**1.9**
		60	2.5	**2.5**	**2.5**	**2.5**	**2.5**	**2.5**	2.7	**2.5**	4/10	**2.5**
		80	3.1	**3.1**	**3.1**	**3.1**	**3.1**	**3.1**	3.3	**3.1**	**3.1**	9/10
		100	3.9	**3.9**	**3.9**	**3.9**	**3.9**	**3.9**	4.0	**3.9**	**3.9**	**3.9**
3	40×40	20	5.1	**5.1**	**5.1**	**5.1**	**5.1**	**5.1**	**5.5**	**5.1**	9/10	**5.1**
		40	9.2	7/10	**9.4**	**9.4**	**9.4**	**9.4**	9.7	9.5	9/10	**9.4**
		60	13.6	*T.O.*	**13.9**	**13.9**	**13.9**	14.0	14.0	14.0	5/10	**13.9**
		80	18.7	*T.O.*	**18.9**	**18.9**	**18.9**	19.1	19.8	19.1	*T.O.*	**18.9**
		100	22.1	*T.O.*	**22.3**	**22.3**	**22.3**	22.6	23.6	22.6	*T.O.*	**9/10**

**Table 11:** Comparison of the solutions obtained by the different approaches for the *cgcut*, *ngcut*, and *beng* instances without rotations.

Class	*n*	# inst	LB*	P&C	BRKGA	GVND	SCH	GLS	TS3	BBMV
*cgcut*	16-62	3	9	**9**	**9**	**9**	**9**	**9**	**9**	**9**
*ngcut*	7-22	12	2.67	**2.67**	**2.67**	**2.67**	**2.67**	**2.67**	3	**2.67**
*beng* 1-8	20-120	8	6.75	**6.75**	**6.75**	**6.75**	6.88			7/8
*beng* 9-10	160-200	2	6.5	**6.5**	**6.5**	**6.5**				

Table 10 shows that the P&C methodology is efficient since it validates the optimality of the solutions obtained by the metaheuristic methods, and for several instances, it finds the optimal solutions for all the instances of the class while the other exact methods were not able to. For Class 3 with 40 items, the P&C is only able to obtain optimal solutions for 7 out of 10 instances. Indeed, the number of variables and constraints for the P&C methodology is too large for these instances. Nevertheless, the P&C approach solves all the instances of Classes 1 and 2 while the BBMV approach or even the DCSP one do not.

The experimental comparison for the *cgcut*, *ngcut*, and *beng* instances is shown in Table 11. The first column indicates the class of the instance, the second column is the number of items to be packed into the bins, and the third column is the total of instances. The fourth column is the best-known lower-bound (average of all instances of the class). The rest of the columns indicate the results obtained by the P&C, BRKGA, GVND, SCH, GLS, TS3, and the BBMV, respectively. No experimental results are provided for these instances for the DCSP methodology.

Since the P&C is an exact method, it finds optimal solutions for instances where the SCH, GLS, TS3, and BBMV could not (*beng 1-8* and/or *beng 9-10*). Therefore, we have demonstrated the performance of the P&C method. The BRKGA and the GVND already were at the optimum value since they matched the lower bound. Recall that the P&C starts with a fixed number of *K* bins, if there is no solution (there is no possible packing all the items in these bins), then *K* is increased, and the covering model is solved again. The process iterates increasing *K* until finding a solution that corresponds to the optimal one.

We now present experimental results for the instances that allow a 90° rotation of the items. The efficiency of the valid inequalities for the ILP model of the set-covering stage of the P&C methodology is similar to the previous section, where no rotations are allowed. Thus, we only present the comparisons of the P&C methodology (enhanced by Eqs (7) and (8)) with the other ones.

Table 12 shows the results for the *bwmv* instances when 90° rotation of the items is allowed. The structure of this table is similar to Table 10, but we only compare ourselves against the BRKGA, HHA-NO(r), HHA-NO(sr), and SVC2BPRF approaches, since they are the only ones that present solutions with rotations by 90° of the items. The lower bounds (LB) for this table were obtained from [40]

**Table 12:** Comparison of the solutions obtained by the different approaches for the *bwmv* instances when 90° rotation of the items is allowed.

Class	*W* × *H*	*n*	LB*	P&C	BRKGA	HHA-NO(r)	HHA-NO(sr)	SVC2BPRF
1	10×10	20	6.6	**6.6**	**6.6**	**6.6**	**6.6**	**6.6**
		40	12.8	**12.8**	**12.8**	13.1	12.9	**12.8**
		60	19.5	**19.5**	**19.5**	19.6	**19.5**	**19.5**
		80	27.0	**27.0**	**27.0**	**27.0**	**27.0**	**27.0**
		100	31.3	**31.3**	**31.3**	31.4	**31.3**	**31.3**
2	30×30	20	1.0	**1.0**	**1.0**	**1.0**	**1.0**	**1.0**
		40	1.9	**1.9**	**1.9**	2	**1.9**	2.0
		60	2.5	**2.5**	**2.5**	**2.5**	**2.5**	**2.5**
		80	3.1	**3.1**	**3.1**	**3.1**	**3.1**	3.2
		100	3.9	**3.9**	**3.9**	**3.9**	**3.9**	4.0
3	40×40	20	4.7	**4.7**	**4.7**	4.8	4.8	**4.7**
		40	9.1	9/10	**9.2**	9.5	9.5	**9.2**
		60	13.2	*T.O.*	**13.4**	13.7	13.7	**13.4**
		80	18.2	*T.O.*	**18.2**	18.6	18.7	18.4
		100	21.5	*T.O.*	**22.0**	22.5	22.5	22.0

We can observe a reduction in the number of bins in contrast with the results of Table 10 (without rotations) and with the lower bound obtained in the literature for the non-rotation case. Also, the P&C could solve the instances up to Class 3 with 20 items. For 40 items, our methodology only solved 9 out of 10. With these results, we have validated that the heuristical solutions of [2] are indeed the optimal ones. Recall that the number of valid positions, and therefore the number of variables in the P&C set-covering formulation can almost double when rotation is allowed. Thus, these instances are harder to solve by our exact methodology.

Table 13, similar to Table 11, shows the comparison results of the P&C methodology for the *cgcut*, *ngcut*, and *beng* instances against the BRKGA (the only approach from Table 9. The rotation of the items decreases the number of bins needed to pack all of them by improving the lower bounds obtained for the non-rotation case. We have again validated that the solutions obtained by the genetic algorithm are indeed the optimal ones for the instances that the P&C could solve.

**Table 13:** Comparison of the solutions obtained by the different approaches for the *cgcut*, *ngcut*, and *beng* instances when 90° rotation of the items is allowed.

Class	*n*	# inst	LB*	P&C	BRKGA
*cgcut*	16-62	3	9	**7.67**	**7.67**
*ngcut*	7-22	12	2.67	**2.5**	**2.5**
*beng* 1-8	20-120	8	6.75	**6.75**	**6.75**
*beng* 9-10	160-200	2	6.5	**6.5**	**6.5**

Usually, one does not have the LB* value at hand (as in the non-rotation case) since it is the best lower bound of many procedures from the literature and an exact algorithm that usually take long computing time. So the P&C methodology is valuable for guaranteeing optimality for small-medium size instances.

## Conclusion

In this study, we present a new two-stage methodology, called *Positions and Covering* (P&*amp*;C), to obtain exact solutions for the two-dimensional bin packing problem (2D-BPP). The first stage is the key-point of the methodology in which, for each item, a set of possible positions that this item can take into the bin is generated in a pseudo-polynomial way. In the second stage, a new set-covering model is solved to select the optimal packing for each bin. By considering the decision version of 2D-BPP and a lower bound on the number of bins for each instance, the P&C methodology verifies if there exists a feasible solution of the instance. If true, then this solution corresponds to the optimal one. Otherwise, the number of bins is increased by one, and the model is solved again until finding a feasible solution. We propose three sets of valid inequalities and make a comparison between the performance of the new mathematical formulation and the incorporation of each valid inequality.

The P&C methodology was tested using the literature benchmark for the 2D-BPP. Due to the combinatorial complexity of the problem, the method could not solve large instances. Nevertheless, for small and medium sizes, P&C was able to verify that the solutions proposed by other approaches were indeed optimal.

We generalize the P&C to consider the case with rotations of the items by 90°. The set-covering ILP related to this case can have almost the double of variables. In the experimental section, we observed the difficulty for solving these instances. Nevertheless, to the best of our knowledge, the generalized P&C is one of the few exact algorithms to solve the 2D-bin packing with rotations. Therefore, with this approach, we can verify the efficiency of other approximation methodologies at less for small and medium size instances.

Due to the novelty of P&C and its efficiency to solve small and medium instances, a natural research line is to develop a decomposition method like a column generation or branch and price, to solve large instances and reduce the computational times.

An interesting research line is to improve the lower bounds of the set-covering model or to generate new cuts to strengthener the model. Finally, it will be interesting to consider the 2D-strip packing and the 3D-bin packing problems with this approach.
